# Migratory songbirds exhibit seasonal modulation of the oxygen cascade

**DOI:** 10.1242/jeb.245975

**Published:** 2023-08-29

**Authors:** Catherine M. Ivy, Christopher G. Guglielmo

**Affiliations:** Department of Biology, Advanced Facility for Avian Research, Western University, London, ON, Canada, N6A 3K7

**Keywords:** Control of breathing, Haematology, Muscle morphology, Thrushes, Warblers, Vireos

## Abstract

Migratory flight requires birds to maintain intensive aerobic exercise for many hours or days. Maintaining O_2_ supply to flight muscles is therefore important during migration, especially since migratory songbirds have been documented flying at altitudes greater than 5000 m above sea level, where O_2_ is limited. Whether songbirds exhibit seasonal plasticity of the O_2_ cascade to maintain O_2_ uptake and transport during migratory flight is not well understood. We investigated changes in the hypoxic ventilatory response, haematology and pectoralis (flight) muscle phenotype of 6 songbird species from 3 families during migratory and non-migratory conditions. Songbirds were captured during southbound migration in southern Ontario, Canada. Half of the birds were assessed during migration, and the rest were transitioned onto a winter photoperiod to induce a non-migratory phenotype and measured. All species exhibited seasonal plasticity at various stages along the O_2_ cascade, but not all species exhibited the same responses. Songbirds tended to be more hypoxia tolerant during migration, withstanding 5 kPa O_2_ and breathed more effectively through slower, deeper breaths. Warblers had a stronger haemoglobin–O_2_ affinity during autumn migration (decrease of ∼4.7 Torr), while the opposite was observed in thrushes (increase of ∼2.6 Torr). In the flight muscle there was an ∼1.2-fold increase in the abundance of muscle fibres with smaller fibre transverse areas during autumn migration, but no changes in capillary:fibre ratio. These adjustments would enhance O_2_ uptake and transport to the flight muscle. Our findings demonstrate that in the O_2_ cascade there is no ideal migratory phenotype for all songbirds.

## Teaser

**Highlighted Article:** Migratory songbirds exhibit seasonal flexibility along the oxygen cascade, which enhances oxygen uptake and movement to the flight muscles during migratory flights.

## INTRODUCTION

Migration allows animals to maximize fitness in response to seasonal changes in resources and is typically associated with movement between breeding areas and non-breeding areas (Dingle, 2014). Migratory flight is considered a feat of great endurance, as birds are required to maintain exercise for many hours or days, at an intensity equivalent to twice the maximal aerobic capacity of running mammals (Butler, 1991). Some birds that demonstrate this extreme ability are bar-tailed godwits (*Limosa lapponica*), which make a single flight of up to 11,700 km from Alaska to New Zealand (Gill et al., 2005) and blackpoll warblers (*Setophaga striata*), which also complete one long flight during their southbound migration, travelling as far as 2540 km in 62 h (DeLuca et al., 2015). Recently, tracking studies have shown that during migratory flight some shorebirds (*Limosa limosa*) and songbirds (*Acrocephalus arundinaceus*) will ascend from ground or sea level to altitudes of 3000–5000 m or greater above sea level (a.s.l.) for 12 h periods (Senner et al., 2018; Sjöberg et al., 2021). It is suggested that migratory flight at high altitude allows birds to find crucial tail winds and refuge from predators (Senner et al., 2018; Sjöberg et al., 2021) but at the expense of a low oxygen (hypoxic) and hypobaric environment. How these birds are able to maintain such intense aerobic exercise for so long under hypobaric–hypoxic conditions, especially after spending a typical breeding season closer to sea level, is not well understood.

Our understanding of high-altitude migration is based primarily on the bar-headed goose (*Anser indicus*), a high-altitude native which migrates over the Himalayan mountains to breeding grounds on the Tibetan Plateau (4800 m a.s.l.) (Meir and Milsom, 2013; Scott and Milsom, 2007). This species has evolved physiological adaptations important for life and migration in hypoxia that maintain O_2_ uptake and supply to tissues. Some enhancements to the O_2_ cascade, which comprises ventilation, pulmonary O_2_ diffusion, circulation, and tissue O_2_ diffusion and utilization, include increased ventilation, increased haemoglobin (Hb)–O_2_ binding affinity, and increased proportions of sub-sarcolemmal mitochondria in flight muscle (Meir and Milsom, 2013; Scott and Milsom, 2007; Scott et al., 2009). All of these traits act to enhance O_2_ movement from the lungs to the muscle mitochondria. A recent study also showed that in flights lasting 1–7 min in normobaric hypoxia, bar-headed geese maintained heart rate but decreased venous O_2_ partial pressure and reduced metabolic rate by 35%, suggesting increased muscle O_2_ extraction efficiency and selective metabolic suppression of non-essential organs (Meir et al., 2019). Few studies have investigated other bird species flying in hypobaric hypoxia (Tucker, 1968) but most have not focused on O_2_ uptake and extraction. Therefore, whether migratory songbirds exhibit similar responses/modifications along the O_2_ cascade to the bar-headed goose is unknown.

Because most migratory songbirds and shorebirds live at low elevations, in order to fly at high altitude they may either be adapted for hypoxia and/or they may seasonally adjust their physiology for high altitude during migration seasons. Past research suggests that long-distance migratory passerines have increased capillary density in flight muscles compared with short-distance and non-migratory species (Lundgren and Kiessling, 1988), which would be important for maintaining O_2_ supply to flight muscles. However, whether there are adaptations in other aspects of the O_2_ cascade, such as ventilation, in migratory songbirds and shorebirds is unknown. In terms of seasonal adjustments, previous research suggests that songbirds do not exhibit locomotory preparations for migration (Hawkes et al., 2017; Portugal et al., 2011; Zúñiga et al., 2016), although there is evidence that flight muscle hypertrophy occurs (Butler and Turner, 1988; Lindström et al., 2000; Marsh, 1984; Marsh and Storer, 1981; Vézina et al., 2021). There is also no change in heart rate but there may be increases in cardiac stroke volume, in preparation for migration (Hawkes et al., 2017). Additionally, seasonal increases in haematocrit (Piersma et al., 1996) have been reported before and during migration, which would help maintain O_2_ supply to mitochondria in hypoxia. Enhancing O_2_ uptake is also beneficial for maintaining lipid oxidation during the migratory season, as fat is a primary fuel source for migrating birds. Lipid-oxidation capacity in flight muscle has been shown to increase during the migratory season further supporting possible modifications in O_2_ uptake along the O_2_ cascade (Guglielmo, 2018; Guglielmo et al., 2002; Saunders and Klemm, 1994). Therefore, whether songbirds exhibit seasonal flexibility in O_2_ uptake and transport to support migratory flight at high altitude requires further investigation.

The objective of our study was to investigate whether songbirds exhibit seasonal flexibility in the O_2_ cascade that is beneficial for migratory flight at high altitude. Migratory songbirds (Order Passeriformes) are an excellent group for examining the general patterns of variation across families because of the various migratory distances within families (e.g. some migrating within North America and some travelling to South America) and variation in body size among families. Vireos (Vireonidae), warblers (Parulidae) and thrushes (Turdidae) were identified as candidate families for this study because of their common migratory route through southern Ontario, Canada and various migratory distances within each family. This allowed us to use a paired design within each family to assess whether migration distance (within North America or to South America) would influence seasonal flexibility: warbling vireos (*Vireo gilvus*), myrtle yellow-rumped warblers (*Setophaga coronata*) and hermit thrushes (*Catharus guttatus*) all migrate within North America, while red-eyed vireos (*Vireo olivaceus*), blackpoll warblers (*Setophaga striata*) and Swainson's thrushes (*Catharus ustulatus*) all migrate to South America. Previous study of myrtle yellow-rumped warblers has shown that they increase flight-muscle oxidative capacity during migration (Dick, 2017), which would enhance O_2_ utilization in the muscle and would be beneficial for migratory flight. Whether other aspects of the O_2_ cascade also exhibit seasonal flexibility in this species has not been studied. Here, we aimed to test the hypothesis that O_2_ uptake and transport are greater in songbirds in the migratory state than in the non-migratory state as a result of seasonal acclimatization. We examined the breathing, haematology and flight-muscle physiology of 6 species of songbirds during the autumn migration and non-migratory conditions to test this hypothesis.

## MATERIALS AND METHODS

### Birds and experimental design

Juvenile songbirds were caught at Long Point, Ontario, Canada, a stopover point during their southbound migration. Myrtle yellow-rumped warblers [*Setophaga coronate* (Linnaeus 1766)] were caught in September 2020 (*N*=16) for haematology and muscle analysis, and in September 2021 (*N*=14) for hypoxic ventilatory responses. Warbling vireos [*Vireo gilvus* (Vieillot 1808), *N*=12], red-eye vireos [*Vireo olivaceus* (Linnaeus 1766), *N*=11], blackpoll warblers [*Setophaga striata* (Forster 1772), *N*=15], hermit thrush [*Catharus guttatus* (Pallas 1811), *N*=16] and Swainson's thrush [*Catharus ustulatus* (Nuttall 1840), *N*=16] were caught in August and September 2021. All birds were housed at the Advanced Facility for Avian Research (London, Ontario, Canada) in free-flight aviaries, and fed a house-made agar-based diet (Dick and Guglielmo, 2019) supplemented with mealworms (*Tenebrio molitor*) and unlimited access to water. Initially, birds were kept on a natural autumn photoperiod (12.5 h light:11.5 h dark), with half being tested under the autumn photoperiod after 3–4 weeks in captivity and then euthanized. The other half were transitioned to a short-day winter photoperiod (9 h light:15 h dark) by mid-November to induce a non-migratory phenotype (Dick, 2017). Non-migratory experiments were conducted after birds had been in short days for 90 days. Nocturnal behaviour was assessed with audio recordings during the sampling periods to confirm that migratory birds, and not non-migratory birds, expressed nocturnal migratory restlessness behaviour. Before terminal sampling (described below), animals were weighed and scanned using quantitative magnetic resonance (QMR; Echo-MRI, Echo Medical Systems, Houston, TX, USA) to measure fat and wet lean mass (Guglielmo et al., 2011). Animal capture and study procedures were approved by the University of Western Ontario Animal Care Committee (Protocol 2018-092) and the Canadian Wildlife Service (SC-OR-2018-0256).

### Acute hypoxia responses

Ventilatory and metabolic responses to acute hypoxia were measured using plethysmography and respirometry techniques similar to those used previously for birds and small mammals (Arens and Cooper, 2005; Bech and Mariussen, 2022; Clemens, 1988; Ivy et al., 2020). Songbirds were individually placed, unrestrained, inside a 1 liter (vireos and warblers) or 2 liters (thrushes) experimental chamber with a perch, at room temperature (∼21°C). Birds were supplied with 21 kPa O_2_ (balance N_2_) at 1 litre min^−1^ or 2 litres min^−1^, respectively, and given 20–40 min to adjust to the chamber (when they exhibited a relaxed and stable breathing pattern) before measurements began. Measurements were then recorded for an additional 20 min at 21 kPa O_2_, before being exposed to 20 min stepwise reductions in inspired O_2_ tension (*P*_O_2__), 16, 12, 9, 7 and 5 kPa. Dry incurrent gases were mixed using precision flow meters (Sierra Instruments, Monterey, CA, USA) and a mass flow controller (MFC-2, Sable Systems, Las Vegas, NV, USA), such that the desired PO_2_ was delivered to the chamber at a constant flow rate of 1 litre min^−1^ or 2 litres min^−1^, respectively. Body temperature (*T*_b_) was measured using a fast-reading mouse rectal thermocouple thermometer (RET-3-ISO; Physitemp, Clifton, NJ, USA) inserted briefly into the cloaca at the end of the experiment and exactly 24 h later for normoxic *T*_b_ (this was used as a proxy for the normoxic *T*_b_ at the start of the experiment, which was not measured to prevent stress to the bird).

Breathing and metabolism were measured continuously during the above exposures, with the average values across the last 10 min at each inspired *P*_O_2__ reported. Excurrent air leaving the animal chamber was subsampled at 200 ml min^−1^, analysed for water vapour (RH-300; Sable Systems), dried with pre-baked Drierite, and analysed for CO_2_ and O_2_ fraction (CA-10 and FC-10; Sable Systems). These data were used to calculate rates of O_2_ consumption (*V̇*_O_2__) and CO_2_ production (*V̇*_CO_2__), expressed at standard temperature and pressure (STP), as recommended by Lighton (2008). Chamber temperature was continuously measured with a thermocouple (PT-6; Physitemp). Breathing frequency and tidal volume were measured from changes in pressure between the animal chamber and reference chamber (which arise from the warming and humidifying of the air as it is inspired by the animal), and were detected using a differential pressure transducer (Validyne DP45; Cancoppas, Mississauga, ON, Canada) connected between the two chambers. Breathing frequency was calculated directly from the ventilation-induced pressure oscillations. Tidal volume was calculated using established equations and expressed as STP (Drorbaugh and Fenn, 1955), assuming a constant rate of decline in *T*_b_ (from the *T*_b_ taken 24 h after the experiment and at the end of the acute hypoxia experiment) with declining *P*_O_2__. Total ventilation was determined as the product of breathing frequency and tidal volume, ventilatory equivalent for O_2_ was calculated by dividing total ventilation by *V̇*_O_2__, and pulmonary O_2_ extraction (%) was calculated as *V̇*_O_2__ divided by the product of total ventilation and inspired fraction of O_2_. Since body mass and lean mass changed between seasons for some but not all species, the displayed ventilatory parameters were not corrected for body mass or lean mass, but lean mass was used as a covariate during statistical analysis (see below). Breathing data were acquired using DATAQ Instruments (DI-1100; DATAQ Instruments Inc. Akron, OH, USA) and WinDaq software (v. 1.30.3; DATAQ Instruments) and were analysed using LabChart 8 Reader (ADInstruments, Colorado Springs, CO, USA). Metabolism and temperature were acquired using a UI-2 and Expedata (v. 1.9.27; Sable Systems).

### Haematology

Blood was collected for Hb–O_2_ affinity assays and haematology after a minimum of 24 h recovery from acute hypoxia response experiments. Approximately 20 μl blood was collected from the wing vein for Hb–O_2_ affinity assays. Oxygen dissociation curves were generated at 41°C for all birds using a Hemox Analyzer (TCS Scientific, New Hope, PA, USA) using 10 μl whole blood in 5 ml buffer (50 mmol l^−1^ HEPES, 10 mmol l^−1^ EDTA, 100 mmol l^−1^ KCl, 0.1% bovine serum albumin, and 0.2% anti-foaming agent; TCS Scientific). Red cell O_2_ affinity (*P*_50_, the *P*_O_2__ at which Hb is 50% saturated with O_2_) was calculated using Hemox Analytic Software (TCS Scientific).

Blood for the remaining haematology measurements was collected at time of sampling. Birds were euthanized by decapitation under isoflurane anaesthesia, and blood was collected. Blood Hb content was measured in a microplate spectrophotometer using Drabkin's reagent (Sigma-Aldrich, Oakville, ON, Canada) in accordance with the manufacturer's instructions. Haematocrit was measured by centrifuging blood in heparinized capillary tubes at 12,000 ***g*** for 5 min.

### Immunohistochemistry of the pectoralis muscle

Muscle fibre and capillary densities were examined in the pectoralis muscle of all birds using immunohistochemistry techniques previously described (Čebašek et al., 2009; Nikel et al., 2018). Muscle samples (∼0.5–1 cm^3^) were taken near the middle of the muscle and spanned from the subcutaneous surface to the sternum. Samples were mounted on cork and coated in mounting medium (Cryomatrix; Thermo Fisher Scientific, Waltham, MA, USA), frozen in liquid N_2_-cooled isopentane, and stored at −80°C until sectioning. Samples were sectioned (12 μm) transverse to muscle fibre length in a cryostat at −20°C. Slides were then air-dried and stored at −80°C.

*Griffonia simplicifolia* lectin 1 (GSL), which binds to terminal α-galactosyl groups and has been used successfully to detect capillaries in various mouse tissues (Hansen-Smith et al., 1992; Laitinen, 1987; Nikel et al., 2018), was used as a marker for capillaries. This was accomplished by first fixing slides in acetone, rinsing in phosphate buffered saline (PBS; 0.01 mol l^−1^, pH 7.4), and letting slides dry for 30 min. Slides were then incubated in hydrogen peroxide (0.3%) for 30 min, rinsed in PBS, and incubated in normal goat serum (NGS; 5%) and PBS containing Tween20 (TPBS, 0.05% Tween20). Slides were then rinsed in PBS and incubated in Avidin D solution (15 min, SP-2001, Vector Laboratories, Burlingame, CA, USA), rinsed again in PBS, and incubated with biotin solution (15 min, SP-2001, Vector Laboratories). After rinsing, slides were then incubated in blocking solution (Carbo-Free Blocking Solution, SP-5040, Vector Laboratories) for 30 min at room temperature. Slides were then incubated overnight at room temperature in a humidified chamber in a solution containing fluorescein-labelled GSL (20 μg ml^−1^; FL-11015, Vector Laboratories) and primary antibodies against laminin (to identify muscle bundle boundaries, 1:200 dilution; L-9393; Millipore, Billerica, MA, USA) in HEPES buffer with *N*-acetylgalactosamine (GalNac; 10 mmol l^−1^ HEPES, 150 mmol l^−1^ NaCl, 0.1 mmol l^−1^ CaCl_2_, 1 mmol l^−1^ GalNac, pH 7.5). The following morning, slides were rinsed well in PBS and then incubated in TPBS containing secondary antibodies against the laminin primary antibodies (Alexa Fluor 594, goat anti-rabbit IgG; A11037, Life Technologies, Mississauga, ON, Canada) for 1 h. Sections were then rinsed thoroughly in PBS and mounted with Vectashield (Vector Laboratories). Sections were imaged using a Leica microscope (CTR6500) with Leica Application Suite Advanced Fluorescence imaging software (v.3.2.0.9652).

Stereological methods were used to make unbiased measurements (Egginton, 1990; Lui et al., 2015). Images were collected systematically such that there was an equal representation of images analysed from across the entire muscle cross-section. Sufficient images were analysed for each sample to account for heterogeneity, determined by the number of replicates necessary to yield a stable mean value. All images were manually analysed in ImageJ (v. 1.50i).

### Statistical analysis

Ventilatory and metabolic measurements were analysed using two-factor ANCOVAs to test for the main effects of season (autumn migration versus non-migratory conditions) and *P*_O_2__ (21, 15, 12, 9, 7, 5 kPa O_2_) within each species with lean mass as a covariate. Holm–Šídák *post hoc* tests were used as appropriate to test for pairwise differences within each *P*_O_2__ value. Body mass, haematological and histological measurements were analysed using two-factor ANOVAs to test for the main effects of season (autumn migration versus non-migratory) and migratory distance (within North America versus to South America) within each family (vireo, warbler, thrush), with Holm–Šídák *post hoc* tests used as appropriate. Percentage wet lean and % fat mass were analysed using a two-factor ANOVA to test for the main effects of season and migratory distance, within each family. All statistical analysis was conducted with R, v. 4.2.0 (https://CRAN.R-project.org/package=nlme). All values are reported as means±s.e.m. and a significance level of *P*<0.05 was considered statistically significant.

## RESULTS

### Body mass and composition

**Fig. 1.**
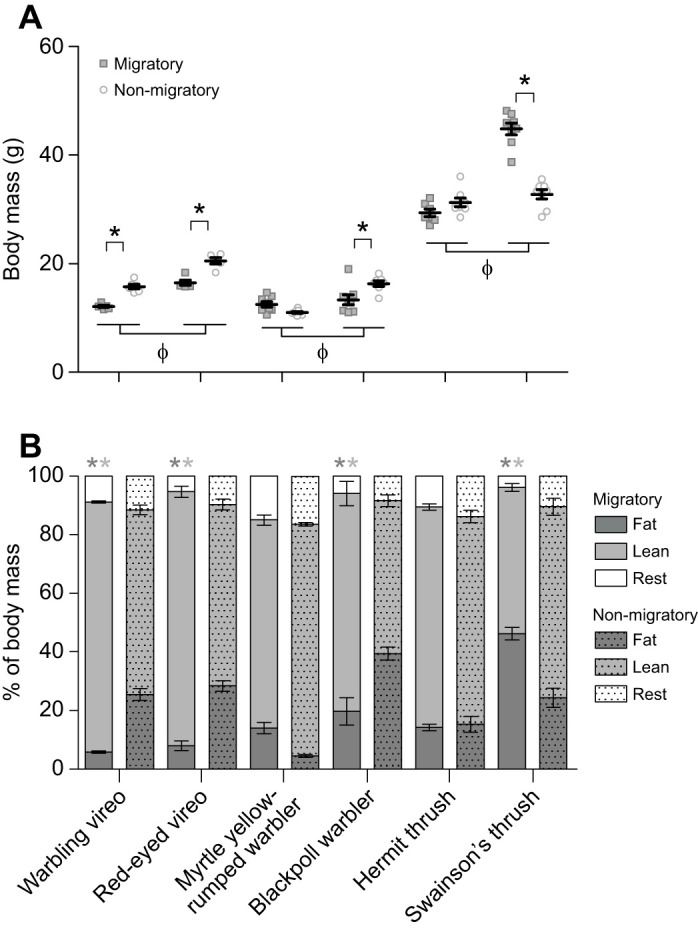
**Body mass and fat measurements are influenced by migration state in most species of songbirds.** (A) Body mass and (B) % fat and % wet lean mass of body mass. Within family, long-distance migrants (red-eyed vireos, blackpoll warblers and Swainson's thrushes) are all heavier than the short-distance migrants (warbling vireos, myrtle yellow-rumped warblers, hermit thrushes). Reductions in body mass during autumn migration are associated with reduced percentages of fat and increased percentages of wet lean mass. Individual values are plotted in A, in addition to mean±s.e.m. φ represents a significant difference between species within a family; * represents a significant difference between migrating and non-migrating conditions within a species, after a two-factor ANOVA within each family; *P*<0.05. *N* for all figures (migratory, non-migratory): warbling vireo=6, 6; red-eyed vireo=6, 5; yellow-rumped warbler=7, 7; blackpoll warbler=8, 7; hermit thrush=8, 8; Swainson's thrush=8, 8.

Body mass and the percentage of wet lean and fat mass were all significantly influenced by season and migratory distance within each songbird family (Fig. 1, Table 1). Songbirds that migrate to South America (red-eyed vireos, blackpoll warblers and Swainson's thrushes) were significantly heavier, regardless of season, compared with their short distance counterparts (warbling vireos, myrtle yellow-rumped warblers and hermit thrushes, respectively). Vireos (warbling and red-eyed) weighed significantly less during autumn migration compared with non-migratory conditions, owing to an increase in the percentage of wet lean mass and a decrease in the percentage of fat mass (Fig. 1). Similar changes in body mass and wet lean/fat percentage were observed in blackpoll warblers, but not in myrtle yellow-rumped warblers (Fig. 1). Myrtle yellow-rumped warblers did not significantly alter body mass with season and only exhibited trends for reduced fat mass (during *post hoc* testing, *P*=0.083) and increased wet lean mass in non-migratory conditions (during *post hoc* testing, *P*=0.073). Hermit thrushes did not significantly alter body mass or wet lean or fat composition between seasons, whereas Swainson's thrushes were significantly heavier during autumn migration and had a greater percentage of fat (Fig. 1).

**Table 1.**
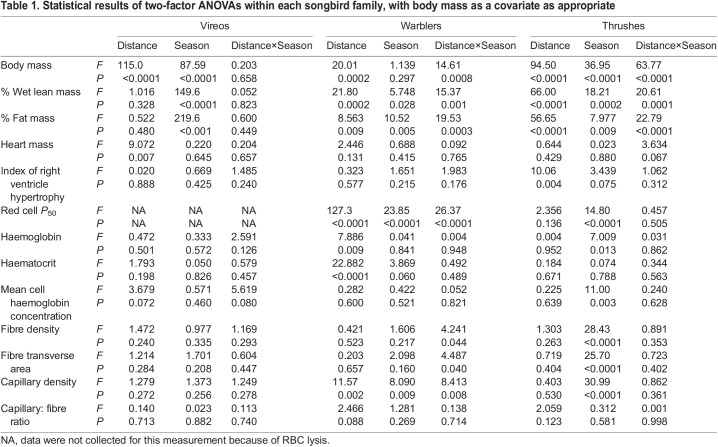
Statistical results of two-factor ANOVAs within each songbird family, with body mass as a covariate as appropriate

### Ventilatory and metabolic responses to acute hypoxia

**Fig. 2.**
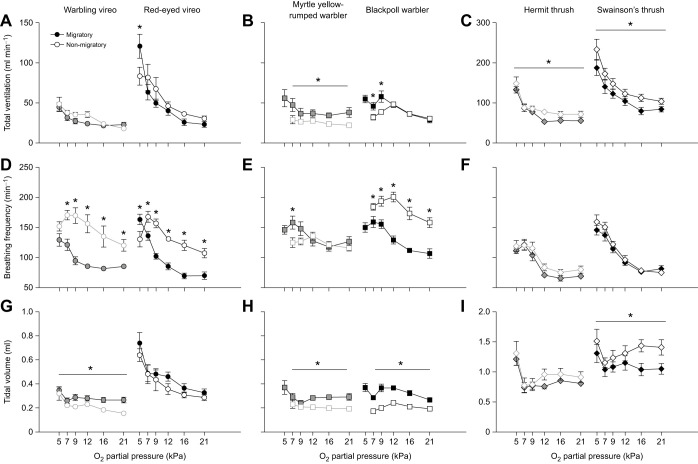
**Breathing responses to progressive hypoxia in vireos, warblers and thrushes during migratory and non-migratory conditions.** Breathing responses to progressive hypoxia (21, 16, 12, 9, 7, 5 kPa O_2_) in vireos (A,D,G), warblers (B,E,H) and thrushes (C,F,I) during migratory (solid symbols) and non-migratory (open symbols) conditions. Increases in total ventilation (A–C) during hypoxia challenge are driven primarily by increases in breathing frequency (D–F) in all species, while tidal volume remains constant (G–I). Grey symbols (graphs on left in each panel) represent short-distance migrants and black symbols (right) represent long-distance migrants. Values are means±s.e.m.; * represents a significant main effect of season (migratory versus non-migratory); * represents a significant pairwise difference between migratory and non-migratory conditions within a *P*_O_2__ level, based on a two-factor ANCOVA within each species; *P*<0.05.

All birds, regardless of season, increased ventilation in response to acute hypoxia challenge (Fig. 2, Table 2). Despite variation in the magnitude of increase in total ventilation in severe hypoxia (1.5- to 5.2-fold above normoxic values; Fig. 2A–C), there was no apparent association with migratory distance. Increases in ventilation were predominantly influenced by increases in breathing frequency (Fig. 2D–F), with minimal changes in tidal volume except in the most severe level of hypoxia (5 kPa O_2_; Fig. 2G–I). Red-eyed vireos were the only birds that significantly increased tidal volume with hypoxia severity (Fig. 2G), in conjunction with breathing frequency (Fig. 2D).

**Table 2.**
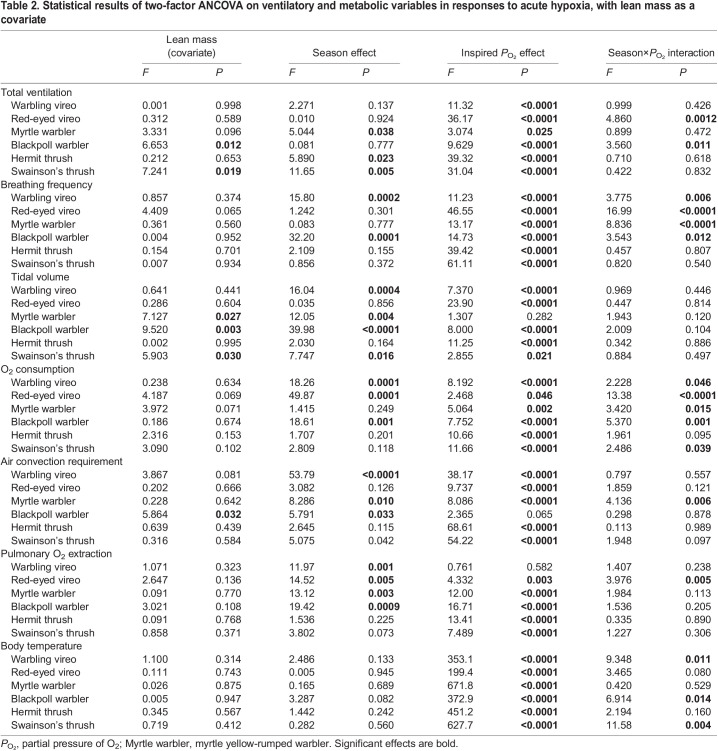
Statistical results of two-factor ANCOVA on ventilatory and metabolic variables in responses to acute hypoxia, with lean mass as a covariate

Hypoxia tolerance and breathing were significantly influenced by season (Fig. 2, Table 2). During autumn migration, all species were able to tolerate stepwise reductions in *P*_O_2__ down to 5 kPa O_2_, but in non-migratory conditions warblers were unable to tolerate this severe level of hypoxia. Vireos and thrushes were able to tolerate 5 kPa O_2_ in non-migratory conditions, although we noted that these species were much more docile upon emergence from the experimental chamber compared with during autumn migration (C.M.I., personal observation). Total ventilation was either maintained between seasons or significantly increased during migration within a species (Fig. 2A–C). Warblers and red-eyed vireos showed increases in ventilation during migration, with yellow-rumped warblers having higher ventilation over all *P*_O_2__ levels during migration (Fig. 2B) and blackpoll warblers and red-eyed vireos showing greater ventilatory responses in the most severe levels of hypoxia (Fig. 2A,B). Breathing frequency was significantly lower in vireos and blackpoll warblers during migration, with breathing frequency showing consistent increases with increasing hypoxia severity (Fig. 2D,E). In non-migratory conditions, breathing frequency appeared to plateau and start to decline at less severe levels of hypoxia compared with migratory conditions, further highlighting declines in hypoxia tolerance. Deeper tidal volumes overall were also observed in warblers and warbling vireos during autumn migration compared with non-migratory birds (Fig. 2G,H). Unlike vireos and warblers, both hermit and Swainson's thrushes had overall significantly higher total ventilation during non-migratory conditions compared with autumn conditions, which was primarily driven by increases in tidal volume and no change in hypoxia tolerance (Fig. 2C,F,I).

**Fig. 3.**
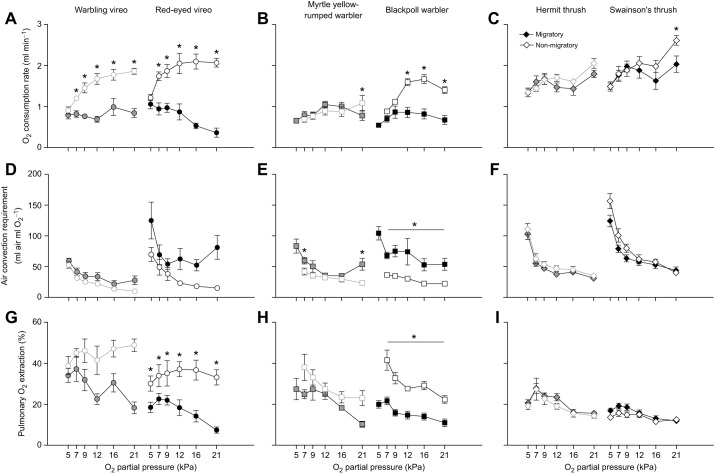
**Breathing and metabolic responses to progressive hypoxia in vireos, warblers and thrushes during migratory and non-migratory conditions.** Oxygen consumption increased during non-migratory conditions in many species (A–C), resulting in reduced air convection requirements (D–F) and greater pulmonary oxygen extraction (G–I) compared with migratory conditions. Grey symbols represent short-distance migrants and black symbols represent long-distance migrants. Values are means±s.e.m.; * represents a significant main effect of season (migratory versus non-migratory); * represents a significant pairwise difference between migratory and non-migratory conditions within a *P*_O_2__ level, based on a two-factor ANCOVA within each species.

**Fig. 4.**
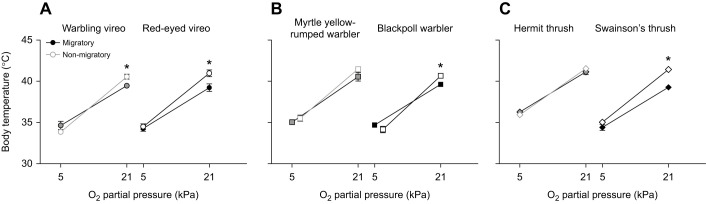
**Body temperature responses to progressive hypoxia in vireos, warblers and thrushes during migratory and non-migratory conditions.** Resting body temperature (normoxia) was significantly lower during the migratory season in most species, but season did not significantly influence the decline in body temperature in hypoxia. Grey symbols represent short-distance migrants and black symbols represent long-distance migrants. Values are means±s.e.m.; * represents a significant pairwise difference between migratory and non-migratory conditions within a *P*_O_2__ level, based on a two-factor ANCOVA within each species.

Regardless of season, *V̇*_O_2__ and body temperature declined with increasing hypoxia severity, while air convection requirement and pulmonary O_2_ extraction increased in hypoxia (Figs 3 and 4, Table 2). Red-eyed vireos were the only species to increase *V̇*_O_2__ with hypoxia severity during autumn migration (Fig. 3A), while all other species maintained *V̇*_O_2__ with declines in the most extreme levels of hypoxia (Fig. 3B,C). Body temperature also declined to a similar extent in all species (∼35°C) in the most severe level of hypoxia (Fig. 4).

Autumn migration reduced *V̇*_O_2__ and the extent of body temperature depression in acute hypoxia challenge in most species (Figs 3 and 4, Table 2). *V̇*_O_2__ was significantly lower during migration in vireos and blackpoll warblers, with minimal change in *V̇*_O_2__ in severe hypoxia compared with normoxic conditions (1.1- to 1.2-fold change) unlike during non-migratory conditions (2.0- to 2.2-fold change) (Fig. 3A,B). This lower *V̇*_O_2__ resulted in higher air convection requirements (Fig. 3D,E) and significantly lower pulmonary O_2_ extraction in red-eyed vireos and blackpoll warblers (Fig. 3G,H). Body temperature was also significantly lower during autumn migration in warbling vireos, red-eyed vireos, and blackpoll warblers compared to non-migratory conditions (∼39.5°C in autumn and ∼41°C in winter), even though body temperature fell to ∼35°C in severe hypoxia in all birds regardless of season (Fig. 4A,B). As with breathing, the thrushes did not show any change in *V̇*_O_2__ with season (Fig. 3C,F,I) and only Swainson's thrush had a significantly lower resting body temperature in normoxia during autumn migration (Fig. 4C).

### The heart and haematological measurements

Heart morphology was not significantly altered with season or migratory distance (Tables 1 and 3). Heart mass did not change with season, although red-eyed vireos had larger hearts compared with warbling vireos (significant main effect of distance within the vireo family), but this difference between species within a family was not present in warblers or thrushes. The index of right ventricle hypertrophy (right ventricle/left ventricle+septum) was not significantly influenced by season, although hermit thrushes had a significantly higher ratio compared with Swainson's thrushes (Tables 1 and 3).

**Table 3.**
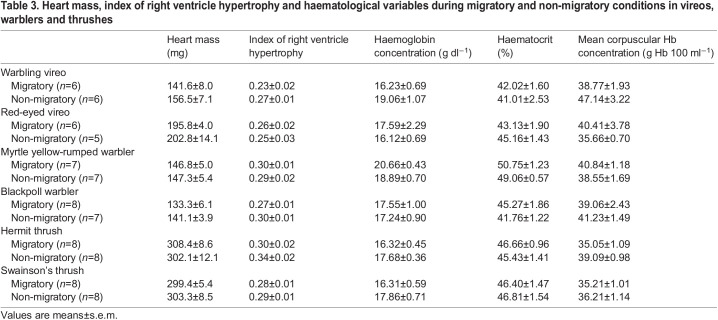
Heart mass, index of right ventricle hypertrophy and haematological variables during migratory and non-migratory conditions in vireos, warblers and thrushes

**Fig. 5.**
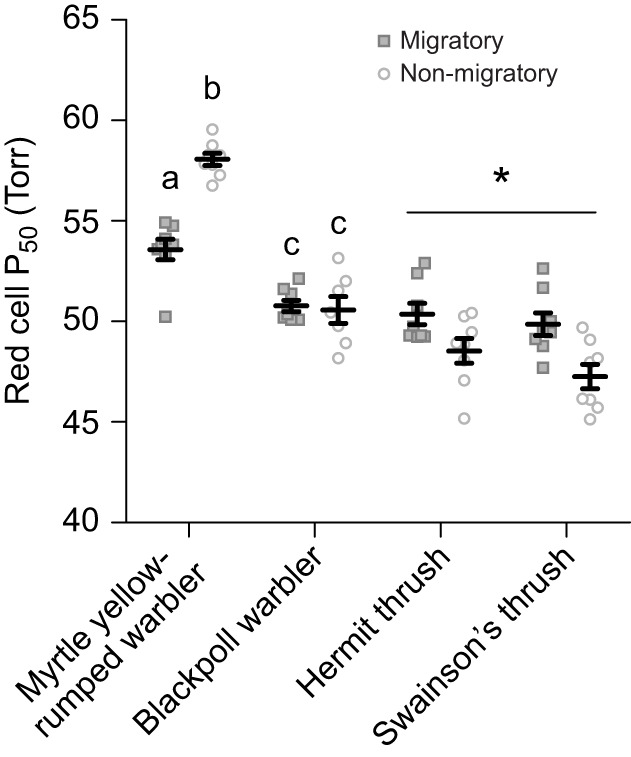
**Haemoglobin-oxygen binding affinity (*P*_50_) in warblers and thrushes during migratory and non-migratory conditions.** Hb–O_2_ affinity was influenced by season in some species. Values are means±s.e.m.; groups that do not share letters within a songbird family represent a significant pairwise difference between season and species; * represents a significant main effect of season within a family after a two-factor ANOVA within each family.

Hb–O_2_ affinity was significantly influenced by season (Fig. 5, Table 1), while haemoglobin concentration, haematocrit and mean corpuscular haemoglobin concentration exhibited no consistent trends (Tables 1 and 3). Hb–O_2_ affinity was significantly stronger (lower *P*_50_) during autumn migration in myrtle yellow-rumped warblers compared with non-migratory conditions, with a *P*_50_ similar to blackpoll warblers, which showed no change in *P*_50_ with season. Thrushes, overall, also significantly altered Hb–O_2_ affinity with season, but in contrast had weaker binding affinity (higher *P*_50_) during migration compared with non-migratory thrushes. Unfortunately, we were not able to measure Hb–O_2_ affinity in vireo red blood cells, as all red blood cells lysed in the buffer used for measuring *P*_50_. Vireos did not exhibit any changes in haemoglobin concentration or haematocrit between species or with season, while myrtle yellow-rumped warblers exhibited higher haemoglobin concentration and haematocrit compared with blackpoll warblers, regardless of season (Tables 1 and 3). Alternatively in thrushes, haemoglobin concentration significantly increased during the winter, but this was not complemented by changes in haematocrit (Tables 1 and 3). No changes in mean corpuscular haemoglobin concentration were observed in vireos or warblers with season or species, with only a significant increase in the non-migratory thrushes (Tables 1 and 3).

### Pectoralis muscle histology

**Fig. 6.**
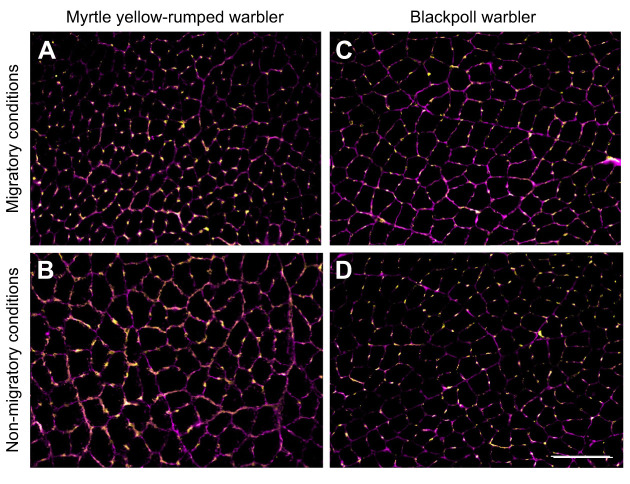
**Representative images of the pectoralis muscle of myrtle yellow-rumped warblers and blackpoll warblers during autumn migration and non-migratory conditions.** Fluorescence immunohistochemistry was used to identify muscle boundaries (laminin, in magenta) and capillaries (*Griffonia simplicifolia* lectin 1, in yellow). Scale bar: 100 μm.

**Fig. 7.**
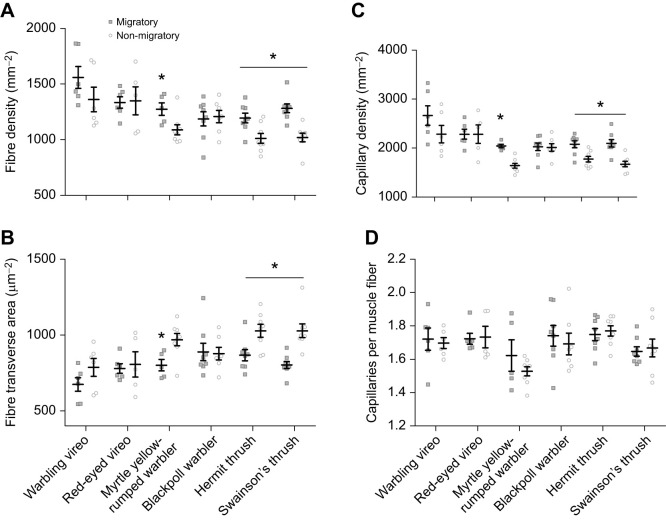
**Fibre and capillary histology of the pectoralis muscle in vireos, warblers and thrushes during migratory and non-migratory conditions.** Fibre density (A), transverse area (B) and capillary density (C) are influenced by season in warblers and thrushes, and mainly in the short-distance migrants (those that stay within North America), but capillary-fibre ratio is not altered with season (D). Values are means±s.e.m.; * represents a significant pairwise difference between migratory and non-migratory conditions within a species; * represents a significant main effect of season within a family after a two-factor ANOVA within each family.

Season and family affected fibre and capillary density in the flight muscle (Figs 6 and 7, Table 1). Fibre density was found to be significantly greater in migrating warbling vireos, myrtle yellow-rumped warblers, hermit thrush and Swainson's thrush, and was associated with smaller fibre transverse areas (Fig. 7A,B). Owing to the smaller fibre size, capillary density was also significantly greater in these species during migration (Fig. 7C), but the number of capillaries per fibre was maintained regardless of season (Fig. 7D). No significant changes in flight muscle morphology were observed with season in red-eyed vireos or blackpoll warblers. Both vireo species had a higher fibre density, with smaller fibre transverse area and higher capillary density compared with the warblers and thrushes studied.

## DISCUSSION

Recent tracking studies have shown songbirds residing at sea level/low altitude can conduct high-altitude flight during migration (Senner et al., 2018; Sjöberg et al., 2021; Williams et al., 1977). The ability of these songbirds to maintain oxygen uptake and movement to the flight muscle in a hypoxic environment is not well understood. Here, we show that songbirds exhibit seasonal flexibility along the O_2_ cascade that enhances O_2_ uptake and movement to the flight muscle during migration. Various species were observed to increase hypoxia tolerance and breathe more effectively to increase O_2_ uptake during autumn migration, with additional modifications in Hb–O_2_ binding affinity and increases in fibre and capillary densities in the flight muscle to enhance O_2_ movement to the muscle. Only changes in flight muscle morphology appeared to be influenced by migratory distance. Although not all species and/or families exhibited all of the same modifications along the O_2_ cascade during autumn migration, these findings highlight the ability of migratory songbirds to exhibit seasonal flexibility in the O_2_ cascade during the migratory season.

### Most songbirds exhibit seasonal flexibility in ventilatory and metabolic responses to hypoxia

All birds, regardless of migratory condition, responded to acute hypoxia with increases in ventilation. Ventilation and breathing pattern in normoxia appear to be similar to those observed in other songbird studies (Arens and Cooper, 2005; Bech and Mariussen, 2022), although studies investigating hypoxic ventilatory responses in songbirds are lacking for comparison. Increases in breathing frequency primarily contributed to increases in total ventilation in hypoxia, which has previously been observed in many species of birds and mammals, with smaller or negligible increases in tidal volume (Fig. 2) (Ivy and Scott, 2017; Ivy et al., 2019, 2020). In contrast, red-eyed vireos exhibited increases in breathing frequency and tidal volume in hypoxia (Fig. 2G). Increases in tidal volume can be beneficial for enhancing O_2_ uptake because increases in tidal volume more effectively increase parabronchial ventilation compared with increases in breathing frequency (Boggs et al., 1984; Ivy et al., 2019; Tenney and Boggs, 1986) but can be more metabolically expensive (Vitalis and Milsom, 1986; York et al., 2017). Many of our species exhibited significant increases in tidal volume in the most severe level of hypoxia (5 kPa O_2_) and this is most likely to enhance parabronchial ventilation in such severe hypoxia.

Seasonal flexibility was observed in breathing pattern in vireos and warblers, but not in thrushes. Migrating vireos and warblers took slower and/or deeper breaths during autumn migration compared with non-migratory conditions. These changes in breathing pattern with migratory condition support those previously observed in other studies investigating seasonal changes in breathing with temperature in songbirds (Arens and Cooper, 2005; Bech and Mariussen, 2022). Although these seasonal changes in breathing pattern do not always correspond with changes in total ventilation, as observed with vireos, the slower deeper breaths during autumn migration would be important for enhancing effective ventilation and bringing in more O_2_ with each breath. Additionally, non-migratory vireos and warblers were less hypoxia tolerant, with warblers unable to withstand 5 kPa O_2_ and vireos exhibiting declines in breathing frequency at the most severe level of hypoxia compared with vireos at the autumn migration (Fig. 2D). In contrast, hermit thrushes did not exhibit any significant changes in breathing frequency or tidal volume with season, while Swainson's thrushes counterintuitively increased tidal volume in non-migratory conditions.

We also observed seasonal flexibility in oxygen consumption and body temperature in vireos and warblers, but not thrushes. Previous studies on wintering birds support the increased *V̇*_O_2__ and pulmonary O_2_ extraction we observed in vireos and blackpoll warblers during non-migratory conditions compared with autumn migration (Fig. 3) (Arens and Cooper, 2005; Bech and Mariussen, 2022). These increases appeared to be associated with increased resting body temperature in normoxic conditions, as warbling vireos, red-eyed vireos and blackpoll warblers were all warmer during non-migratory conditions compared with autumn migration (Fig. 4). A higher metabolic rate during the winter in songbirds is thought to be important for enhancing their metabolic capacity during the colder months of the year (Bech and Mariussen, 2022; McKechnie et al., 2015), whereas this lower metabolic rate and body temperature during autumn migration might be important for reducing metabolic costs during the migratory season. Our songbirds exhibited an increase in body temperature during non-migratory conditions similar to those reported in a boreal passerine, the non-migratory great tit (*Parus major*) (Bech and Mariussen, 2022). These findings are surprising since the songbirds we studied here typically migrate to temperate climates and were not acclimated to cold conditions, but could suggest that these birds may winter at higher altitudes (Williamson and Witt, 2021). Why myrtle yellow-rumped warblers did not show these changes in metabolism and body temperature requires further investigation. Additionally, thrushes did not alter metabolism, and only hermit thrushes did not alter body temperature with season, which could be due to their larger size compared with vireos and warblers.

The seasonal plasticity observed in pulmonary O_2_ extraction in red-eyed vireos and blackpoll warblers highlights that there may be changes in pulmonary O_2_ diffusion and/or O_2_ circulation. Although previous studies support seasonal changes in pulmonary O_2_ extraction (Arens and Cooper, 2005; Bech and Mariussen, 2022), the mechanism resulting in this change has not been well studied in a seasonal context. Andean geese (*Chloephaga melanoptera*), which reside and migrate at high altitude in the Andean Altiplano, have a blunted ventilatory response to hypoxia compared with the bar-headed goose, but overall higher pulmonary O_2_ extraction (Lague et al., 2017). This increased O_2_ extraction is associated with a high respiratory surface area and vascularization (Maina et al., 2017), paired with a strong Hb–O_2_ binding affinity (Natarajan et al., 2015). In our blackpoll warblers we observed an overall stronger Hb–O_2_ affinity compared with yellow-rumped warblers that was not altered with season (discussed below), suggesting that there could be seasonal changes in lung morphology or cardiac responses. Alternatively, owing to the overall stiffer structure of the avian lung compared with the mammalian lung, seasonal changes in respiratory surface area and vascularization are probably less likely to occur, suggesting that changes in O_2_ circulation through cardiac output may be more likely (Hawkes et al., 2017). Future research investigating the lung morphology and the cardiovascular system of migratory songbirds with changes in migratory state are required.

### O_2_ circulation did not show consistent seasonal flexibility across species

The ability to extract O_2_ from the lungs during flight is important for maintaining O_2_ transport to the flight muscle. Based on our hypothesis, we predicted Hb–O_2_ affinity would be stronger during the migratory season, to enhance O_2_ extraction from the lungs. An increased Hb–O_2_ affinity is observed in the bar-headed goose (Black and Tenney, 1980; Jessen et al., 1991; Meir and Milsom, 2013; Natarajan et al., 2018; Weber et al., 1993), which aids in maintaining a higher arterial O_2_ saturation despite a reduced inspired *P*_O_2__ (Scott and Milsom, 2006). Our prediction was supported in myrtle yellow-rumped warblers, which significantly increased Hb–O_2_ affinity during autumn migration (Fig. 5). In contrast, blackpoll warblers did not show seasonal flexibility in Hb–O_2_ binding affinity, and had stronger affinity overall compared to myrtle yellow-rumped warblers, which may be important for their long-distance migratory flights. Thrushes also showed seasonal flexibility in Hb–O_2_ affinity, with reduced Hb–O_2_ affinity during autumn migration (Fig. 5). Reduced Hb–O_2_ affinity would be beneficial for unloading at the muscle, and given the efficiency of the avian lung, probably results in minimal reductions in O_2_ uptake at the lung. Warbling and red-eyed vireos were sampled for Hb–O_2_ affinity measurements, but their red blood cells burst in the buffer solution, unlike warbler and thrush blood. Hb–O_2_ affinity has not been well studied in migratory species, so whether other species show similar seasonal responses is unknown. No additional seasonal changes in haematology were observed, in contrast to previous findings of increased haemoglobin concentration and haematocrit during migration in songbirds (Krause et al., 2016). Hb–O_2_ affinity could be altered in these species by changes in inositol pentaphosphate concentrations (an allosteric modifier; Landini et al., 2013) or the proportions of HbA/HbD, as HbD is known to have a stronger binding affinity than HbA (Storz, 2016). The varying seasonal responses in haematology highlight that there does not appear to be a single optimal phenotype at this step of the O_2_ cascade in migratory songbirds.

### Migratory distance influences seasonal flexibility in flight muscle morphology

We observed seasonal flexibility in the flight muscle of migratory songbirds. Our data show that muscle fibre transverse area is significantly smaller during autumn migration compared with non-migratory conditions in two of our short-distance migrants (myrtle yellow-rumped warblers and hermit thrush, with a similar visual trend in warbling vireos), similarly to willow warblers in Europe (Lundgren and Kiessling, 1988). Myrtle yellow-rumped warblers and hermit thrush increased the number of fibres and had smaller fibre transverse areas during autumn migration compared with non-migratory conditions. Capillary density also increased during autumn migration, but when calculated per muscle fibre, remained constant regardless of season. Although larger fibres would provide greater power output during migratory flight, they may come at a cost for smaller passerines, resulting in them favouring fibres with smaller transverse areas that would enhance O_2_ and fuel diffusion into and out of the muscle to power flight (Kinsey et al., 2011). Larger muscle fibres during winter would be important for increasing heat generation, as the pectoralis muscle is the main heat-generating organ in small passerines (Swanson and Vézina, 2015). These seasonal changes could be the result of changes in muscle fibre phenotype, as few studies have investigated seasonal changes in muscle fibre phenotype in migratory songbirds.

Long distance migratory vireos and warblers did not show seasonal flexibility in flight muscle morphology. Red-eyed vireos and blackpoll warblers maintained muscle fibre density and fibre transverse area regardless of season, at values equivalent to or slightly higher than their non-migratory short-distance counterparts (warbling vireos and myrtle yellow-rumped warblers, respectively). These findings suggest that the distance these passerines travel may require them to maintain slightly larger muscle fibres to power their southbound migration. This may be particularly true for the blackpoll warbler, which can travel as far as 2540 km in 62 h (DeLuca et al., 2015). In contrast to red-eyed vireos and blackpoll warblers, Swainson's thrush did show seasonal flexibility in muscle morphology similarly to hermit thrushes, which may be due to their larger size. This plasticity in Swainson's thrushes highlights that there are many ways that long-distance migratory songbirds can modulate their flight muscle.

Our study allowed us to assess whether there were coordinated changes along the O_2_ cascade that would maintain O_2_ supply to the flight muscle during migratory flight. The concept of symmorphosis proposes that structural design is optimized to match, but not exceed, functional demands and that each step in the O_2_ cascade has an equal capacity to support changes in O_2_ flux (Weibel et al., 1981, 1991). We found that there were no consistent strategies during autumn migration among species, and that not all steps of the cascade were altered. For example, myrtle yellow-rumped warblers significantly increased Hb–O_2_ affinity and decreased fibre transverse area during autumn migration, which would be important for moving O_2_ from the lungs to the flight muscle, but showed no changes in breathing. Given that pulmonary ventilation is responsible for O_2_ uptake, it is intriguing that the succeeding steps in the cascade exhibited plasticity while breathing did not. This could suggest that the breathing step of the cascade may be effective enough for this species during migratory flight, or that there may be seasonal changes with pulmonary O_2_ diffusion that do not require changes in breathing. In contrast, blackpoll warblers only exhibited seasonal plasticity in breathing and none of the following steps in the cascade, although they had an overall stronger Hb–O_2_ affinity. A lack of seasonal plasticity in the following steps of the cascade could be the result of genotypic adaptations important for multi-day migratory flights to South America, while changes in breathing are only important seasonally to support the increased O_2_ demands from the flight muscles. The lack of plasticity in some steps of the cascade within a species could highlight that not all steps of the O_2_ cascade need to be altered to support increased O_2_ demands, as has been observed in artificial selection experiments and theoretical modelling studies (Gonzalez et al., 2006; Henderson et al., 2002; Kirkton et al., 2009; Scott and Milsom, 2006; Wagner, 1996).

A limitation to our study is the amount of time our birds spent in captivity. Our measurements on migratory birds were made three to four weeks after capture because of equipment delays and AFAR quarantine procedures. Differences in measurements between long-distance and short-distance migrants may therefore be exaggerated in migratory, and possibly non-migratory, conditions. For example, long-distance migrants appeared to be better at replacing fat mass during this ‘stopover duration’ (3–4 week period before experiments) compared with short-distance migrants and then did not use this fat mass to finish fuelling their southbound migration before entering non-migratory conditions at our facility (Fig. 1A). Our long-distance migrants therefore may have maintained a higher fat mass when entering non-migratory conditions compared with what is typically observed in the wild. We therefore used lean mass as a covariate with our ventilatory and metabolic measurements, as total body mass would have been misrepresentative.

In conclusion, we observed seasonal plasticity in migratory songbirds during autumn migration that enhances O_2_ uptake and movement to the flight muscle that would be beneficial during high-altitude migratory flight. Although the plasticity we observed would be beneficial for migratory flight, regardless of altitude, our findings highlight the importance of testing the hypoxia tolerance of these birds during flight at simulated altitudes. We observed that not all migratory songbirds used the same strategy for enhancing O_2_ uptake and movement, but there were similarities, such as more effective breathing, changes in Hb–O_2_ affinity, and modulation in pectoralis fibre density and fibre transverse area. Additionally, species size likely plays an important role in the degree of seasonal modulation observed along the O_2_ cascade, as many studies focus on passerines that are 35–40 g or larger in size, whereas our study ranged from 12 to 45 g. More studies investigating seasonal plasticity in a range of passerine sizes would provide greater insight into this finding. Furthermore, we focused on migratory species in this study, whether resident species also exhibit hypoxia tolerance and modifications along the O_2_ cascade is yet to be determined.
